# Beyond the Limits: How Is Spectral Flow Cytometry Reshaping the Clinical Landscape and What Is Coming Next?

**DOI:** 10.3390/cells14130997

**Published:** 2025-06-30

**Authors:** Kamila Czechowska, Diana L. Bonilla, Adam Cotty, Amay Dankar, Paul E. Mead, Veronica Nash

**Affiliations:** 1 Metafora Biosystems, 75014 Paris, France; kamila.czechowska@metafora-biosystems.com; 2 Cytek Biosciences Inc., Fremont, CA 94538, USA; dbonilla@cytekbio.com; 3 KCAS Bio, Lower Gwynedd, PA 19002, USA; adam.cotty@kcasbio.com; 4 GSK, Collegeville, PA 19426, USA; amay.x.dankar@gsk.com; 5 Department of Pathology, St Jude Children’s Research Hospital, Memphis, TN 38103, USA; paul.mead@stjude.org

**Keywords:** spectral flow cytometry, clinical flow cytometry, immune profiling, immune monitoring, clinical trials, biomarkers, diagnostic flow cytometry, data standardization, high-dimensional cytometry, high-dimensional analysis

## Abstract

Spectral flow cytometry has revolutionized traditional single-cell profiling to a new era of high-dimensional analysis, allowing for unprecedented deep phenotyping and more precise cell characterization, thereby significantly enhancing our multiplexing capability. The recent application of this technology in clinical settings has been redefining the landscape of clinical diagnostic panels and immune monitoring, particularly for hematologic malignancies, immunological disorders, and drug discovery. Emerging technologies like ghost cytometry, LASE, and imaging flow cytometry are advancing cytometry by improving sensitivity, throughput, and spatial resolution. In this review, we discuss the requirements, challenges, and considerations for spectral applications in clinical diagnostic laboratories and pharmaceutical/contract research organization (CRO) settings. We discuss how these recent innovations are set to push the boundaries of diagnostic accuracy and analytical power, heralding a new frontier in clinical cytometry with the potential to dramatically enhance patient care and treatment outcomes.

## 1. Introduction

**Figure 1:**
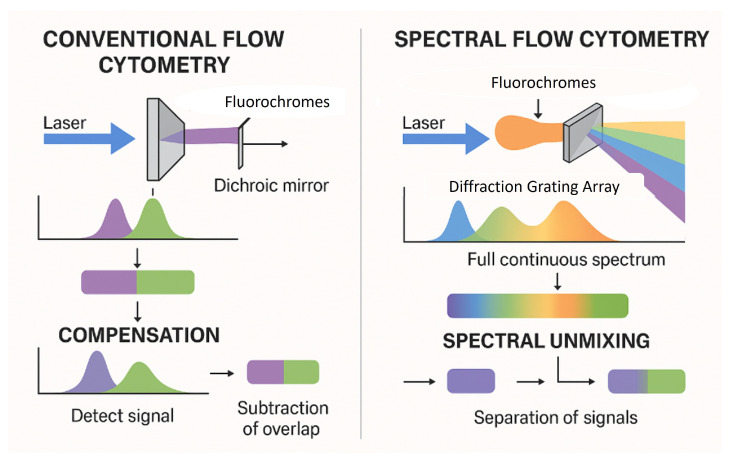
Comparison of conventional vs. spectral flow cytometry.

The evolution from conventional flow cytometry (CFC) to spectral flow cytometry (SFC) represents a significant technological advancement in the field, guided by the need for deeper and more precise cellular characterization. In CFC, each laser line enables the collection of signals by a certain number of detectors (from two to six, never just one) and it is limited to measuring only the peak emission of each fluorochrome. Its ability to multiplex dyes with overlapping emission spectra in the same assay is restricted, and it is complicated by compensation procedures. In contrast, SFC enables both broad and deep phenotyping by using multiple detectors to capture the entire fluorescence emission spectrum for each fluorochrome, across multiple laser lines. This allows for more precise signal unmixing, even between dyes with highly overlapping peak emissions, and permits the simultaneous analysis of a greater number of parameters within a single tube., [1,2] (Figure 1).

Software accompanying spectral technologies further enhance this capability by characterizing and extracting autofluorescence (AF) signals using the same linear unmixing algorithms employed in fluorochrome identification [3,4,5]. Extraction of AF enhances cell characterization and minimizes background noise in comparison to CFC [6], and together with the integration of more sensitive detectors into newer technologies, has significantly enhanced the resolution of cell populations in multiparametric assays, thereby improving the overall accuracy and clarity of the results. Clinically, the enhanced capabilities of SFC support more comprehensive diagnostic panels and refined monitoring of disease states, particularly in hematologic malignancies and immunological disorders. The integration of SFC into clinical practice promises an improved efficiency in disease diagnosis, prognosis, and monitoring. This sets the stage for more personalized and effective therapeutic interventions. As demonstrated by Brestoff et al. (2023) and Soh KT et al. (2022), in addition to increasing the number of markers that can be detected with a high resolution, SFC offers several other advantages [2,7]. These include a reduced sample consumption, decreased need for duplicating markers across multiple tubes (panel backbone markers), and minimized reliance on inferences during data analysis [2].

This review provides a comprehensive look at the current state of SFC, discussing its advantages, challenges, and future directions. Within clinical contexts, we highlight key applications, and the practical challenges associated with its implementation. Finally, we discuss emerging tools and approaches that could further enhance its clinical utility while highlighting the evolving role it is playing in both translational and diagnostic cytometry.

## 2. Spectral Flow Cytometry’s Use in Clinical Endeavors

The value of SFC in drug discovery and clinical analysis has been increasingly recognized for the transformative potential of its applications in panels used in biomarker discovery and diagnostics. These panels enable monitoring and analysis of complex samples, with more enhanced cell characterization capabilities in comprehensive panels. This section explores how SFC is reshaping clinical trials, particularly after its adoption by pharmaceuticals (Pharma) and contract research organizations (CROs), as well as its tremendous impact on clinical diagnostics.

### 2.1. Pharma and CROs: Enhancing Pharmacodynamic Biomarker Monitoring and Discovery

Therapeutic resistance and heterogeneous patient responses in hematologic malignancies underscore the critical need for robust biomarkers to guide treatment selection and predict clinical outcomes. Biomarkers enable a stratification of patients based on their likelihood of response, thereby informing therapeutic decisions and facilitating early interventions. In the context of CD19-directed CAR-T cell therapies, spectral flow cytometry (SFC) has emerged as a powerful tool for monitoring treatment efficacy, tracking CAR-T cell kinetics, and evaluating the measurable residual disease (MRD) [8].

While CD19 CAR-T therapy achieves high initial response rates in B-cell malignancies, relapse remains a common challenge. High-dimensional analysis using SFC permits a simultaneous assessment of CAR-T products, residual disease, and the immune context in a single tube, facilitating real-time clinical insights and immune monitoring endpoints [9]. Although no approved biomarker currently exists for patient selection, recent studies employing high-parameter SFC have identified cellular phenotypes associated with a therapeutic response. For instance, Denlinger et al. (2024) reported an enrichment of PD-1^+^ CD8^+^ CAR-T subsets in responders with lymphoma, while Zhang et al. (2024) observed that CAR-T infusion products enriched in CCR7^+^ early-memory cells and exhibiting low CD39 expression, were predictive of favorable outcomes in chronic lymphocytic leukemia (CLL) [10,11]. Similarly, Odak et al. (2022) demonstrated that distinct phenotypic profiles in infusion products correlated with the toxicity risk [12]. These findings collectively highlight the value of multiparametric cytometry in delineating response and resistance mechanisms in cellular immunotherapy. In the broader immuno-oncology landscape, monitoring T-cell exhaustion has also proven critical, given its established role in therapeutic resistance [13].

SFC offers substantial advantages for biomarker discovery and immune profiling, particularly in clinical trial settings where precision, reproducibility, and throughput are paramount. Its ability to capture the complete fluorescence spectrum, coupled with sensitive detectors and AF extraction, improves the signal resolution for low-abundance markers. The development of high-parameter panels allows for comprehensive immune characterization that is not feasible using conventional flow cytometry [2,13,14]. Nonetheless, challenges intrinsic to cytometry—such as spillover, spreading error, and AF—persist. These are mitigated in SFC through optimized unmixing algorithms, improved optical configurations, and dynamic AF subtraction. As Jameson et al. (2022) noted, the stain index and population resolution are highly dependent on instrument-specific configurations, while Ferrer-Font et al. (2021) and Pilkington et al. (2024) emphasized that improper AF handling may result in unmixing errors and false-positive events [15,16,17,18]. Roet et al. (2024) further demonstrated that although AF subtraction enhances the resolution, it may increase the spread in negative populations, particularly for fluorochromes overlapping with endogenous fluorescence profiles [19].

SFC is especially well-suited to scenarios involving limited specimen availability, including bone marrow aspirates, pediatric biopsies, and post-chemotherapy samples, where conventional multi-tube panels are impractical. Recent work by Spasic et al. (2024) and Soh et al. (2022) demonstrated the feasibility of deploying high-dimensional panels in low-volume settings [2,20]. Additionally, SFC enhances the utility of archived and cryopreserved specimens. Jensen and Wnek (2021) reported the stability of immunophenotypic markers in whole blood up to 72 h post-collection, while Brestoff (2023) showed that over 35 parameters could be routinely analyzed in archived samples using full-spectrum cytometry [7,14]. Edwards et al. (2023) extended this application by profiling cryopreserved samples from melanoma patients, confirming the platform’s suitability for retrospective immune monitoring and longitudinal biomarker evaluation [21].

### 2.2. Clinical Implementation: MRD and Multi-Parameter Diagnostics

SFC supports the development of comprehensive diagnostic panels for clinical use, offering an unprecedented level of multiplexing within a single assay [20]. This is particularly valuable in hematologic malignancies, where broad immunophenotypic diversity complicates diagnosis and treatment stratification [22]. In the context of MRD detection, SFC allows for high-resolution quantification of leukemic burden, critical for risk assessment and guiding treatment decisions. One-tube assays combining lineage and disease-specific markers eliminate the need for sample splitting and increase sensitivity, a key advantage in post-treatment settings characterized by low cellularity. Chen et al. (2023) validated a 24-color SFC panel for MRD detection in acute myeloid leukemia (AML), demonstrating a sensitivity below 0.02% while preserving marker correlation and improving the resolution of maturation states [23]. In acute B-lymphoblastic leukemia (B-ALL), García-Aguilera et al. (2024) described a 23-color panel that identified CD19-negative leukemic clones—a critical challenge following CD19-targeted therapies [22]. Chatterjee et al. (2024) and Gao et al. (2023) further addressed this issue by designing MRD assays incorporating surrogate B-lineage markers such as CD22, CD24, and CD81, along with lineage-specific markers like CD79a [24,25]. These panels achieved sensitivities below 0.001%, enabling the detection of antigen-loss variants. Ramírez et al. (2024) demonstrated the successful implementation of SFC-based MRD monitoring in pediatric clinical workflows, while Verbeek and van der Velden (2024) underscored the importance of updating MRD strategies to accommodate treatment-induced phenotypic shifts [26,27]. The utility of spectral flow cytometry for MRD detection has been demonstrated across various hematologic malignancies [26,27]. Table 1 summarizes key disease-specific applications, highlighting the marker configurations, achieved sensitivity levels, and clinical advantages of SFC-based MRD assays.

SFC is transforming clinical immunophenotyping and translational research. Its ability to resolve complex cellular phenotypes with high dimensionality, particularly in low-volume and archived samples, provides a robust framework for biomarker discovery, therapy monitoring, and minimal residual disease detection. The continued integration of SFC into clinical practice is expected to enhance the precision of diagnostics and support the next generation of personalized medicine.

## 3. Spectral Flow Cytometry Use in the Clinical Setting: Requirements and Challenges

As the number of parameters increases, so does the complexity of spectral panel design. Key considerations include managing fluorochrome compatibility, optimizing signal-to-noise ratios, and minimizing spectral overlap. Like CFC, SFC requires a careful handling of autofluorescence, which can significantly impact data interpretation. The absence of standardized workflows for panel optimization further complicates assay development.

Rigorous assay validation is essential for clinical deployment of SFC. Current gaps in regulatory alignment include the absence of protocols tailored to the spectral platform. Sensitivity thresholds, reproducibility across platforms, and rare-event detection remain areas in need of refinement. Validation must also encompass software tools used for spectral unmixing and data analysis, which are not yet uniformly regulated. To support clinical adoption, protocols must align with key regulatory standards such as ISO 15189, CLSI guidelines (e.g., EP17-A2, EP05-A3, H62), and CE-IVD or U.S. Food and Drug Administration (FDA) requirements [32,33,34,35,36,37,38,39]. For clinical flow cytometry laboratories that participate in the College of American Pathologists (CAP) accreditation programs, a specific flow cytometry checklist, including sections on proficiency testing, quality management and quality control, and procedures and test systems, is used in conjunction with the ‘All Common and Laboratory General Checklists’ during regular on-site inspections for laboratory compliance.

Instrument setup and ongoing performance monitoring are critical for ensuring data quality in SFC. Unlike CFC, spectral instruments require precise calibration for accurate unmixing and signal detection. Long-term standardization efforts are lacking, making it difficult to compare results across instruments with different laser configurations or from different manufacturers, sites, or time points. Development of harmonized protocols and multicenter reproducibility studies are urgently needed.

The high-dimensional data produced by SFC necessitates robust analysis platforms. Traditional tools may be insufficient to handle the volume and complexity of spectral data. Advanced computational methods, including machine learning (ML) and automated gating, are increasingly important for data interpretation [7]. Standardized validation of these tools is a prerequisite for clinical utility [40].

### 3.1. Clinical Implementation and Use Cases

SFC holds promises for clinical applications such as immunophenotyping and MRD detection. However, variability in instrument calibration and gating strategies can lead to significant discrepancies in clinical results [41]. Standardized protocols are vital to ensure a consistent performance regardless of the technique or setting. Cossarizza et al. (2019) provided foundational guidance for implementing flow cytometry in clinical labs, outlining steps toward regulatory compliance and offering a roadmap for broader adoption [6].

To fully realize the potential of SFC in clinical diagnostics, the field must prioritize validation, standardization, and regulatory alignment. Establishing consensus protocols and robust quality control measures will ensure that SFC becomes a reliable and scalable tool for precision medicine.

### 3.2. Regulatory Requirements

SFC is a powerful clinical diagnostic tool that faces significant regulatory challenges due to stringent compliance requirements imposed by bodies such as the U.S. FDA and the European Medicines Agency (EMA). To ensure safety, efficacy, and quality, the FDA in the United States mandates that any diagnostic device—including those based on SFC—undergo a thorough pre-market approval process involving extensive clinical validation studies that demonstrate reliable detection and quantification of specific cellular markers, particularly in complex applications like MRD detection [34,42,43]. Devices must meet performance standards for sensitivity, specificity, and reproducibility across various conditions. The recent FDA ‘Final Rule’ has clarified that lab-developed tests (LDTs), previously subject to enforcement discretion, are now classified as medical devices under 21 CFR Part 809, initiating a five-stage phaseout policy over the next four years [43]. While a recent summary judgement from the United States District Court, Eastern District of Texas, concluding “that the final rule exceeds FDAs statutory authority” has overturned the ‘Final Rule’ in its current state, it remains to be seen what the regulatory framework will look like for LDTs moving forward.

In Europe, the EMA enforces compliance via the In Vitro Diagnostic Medical Device Regulation (IVDR), fully applicable since May 2022. Like the FDA, the IVDR requires manufacturers to provide clinical evidence supporting a device’s analytical and clinical validity, as well as its risk–benefit profile. However, the IVDR’s emphasis on post-market surveillance—continuous monitoring and reporting of a device’s performance—presents additional challenges.

Meeting these regulatory demands is resource-intensive, requiring large datasets that demonstrate a consistent performance across diverse patient populations and conditions—a particular burden for smaller companies or research institutions. Moreover, as for conventional flow cytometry, the complexity of SFC, including variability in instrument settings, sample preparation, unmixing strategies, and data analysis protocols, can lead to significant outcome differences if not tightly controlled across laboratories. As regulatory frameworks evolve to address emerging risks, manufacturers and clinical laboratories must continuously update their practices to remain compliant. As of 2025, only the Cytek Northern Lights system has been cleared for diagnostic in vitro use in China. No SFC instruments have yet received FDA or IVDR approval for clinical diagnostics in the U.S. or EU.

**Figure 2:**
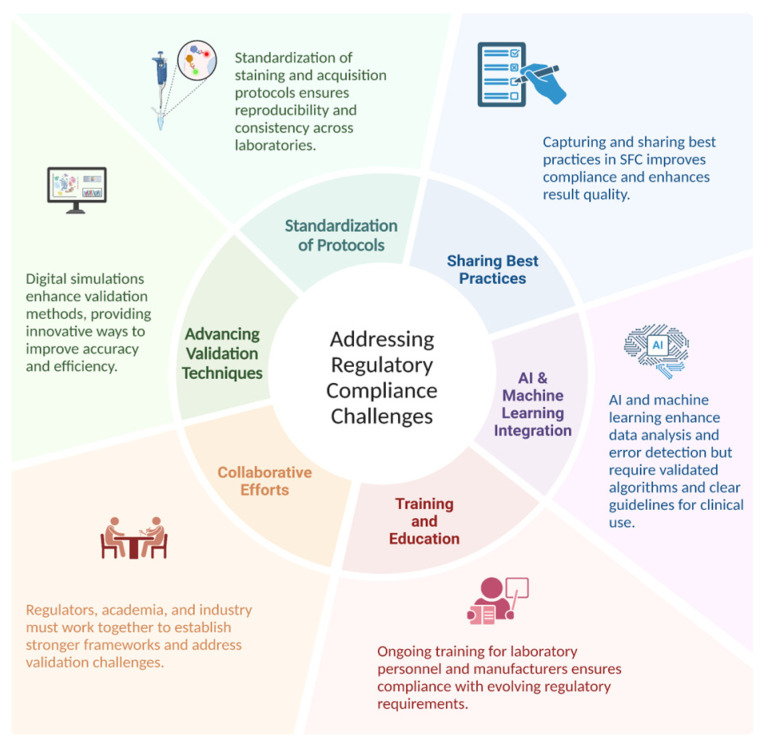
Addressing regulatory compliance challenges.

Improving reproducibility and regulatory compliance in SFC requires standardizing protocols, advancing validation techniques, and fostering collaboration among regulators, academia, and the industry. Enhanced training, sharing best practices, and artificial intelligence (AI) integration for data analysis and error detection can streamline validation processes, though these advances also introduce new regulatory challenges that demand clear guidelines and algorithm validation (Figure 2).

### 3.3. Panel Design Complexity

Spectral technology has greatly enhanced flow cytometry multiplexing by capturing the full spectral fingerprint of each dye across all detectors, rather than relying solely on peak signals. This expanded capability allows for larger panels and increased flexibility in reagent selection; however, it demands meticulous fluorochrome selection to avoid spectral overlap and avoid a loss in resolution due to spectral overlap not due to the choice of fluorochromes, but rather due to inadequate reference controls, as proper controls are needed for accurate unmixing (for a detailed discussion, see Section 3.12 on proper controls). Metrics such as similarity and complexity indices help ensure that unique fluorochromes are chosen. Moreover, dye chemistry plays a critical role: tandem dye can have emission variation across lots, requiring re-titration when a new lot is purchased [44,45,46]. Similarly, the use of polymer-based dyes like Brilliant Violet™ (Brilliant Violet™; BioLegend, San Diego, CA, USA) and RealBlue™ (RealBlue™; BD Biosciences, San Jose, CA, USA) requires specific blocking buffers to prevent non-specific interactions and ensure accurate staining [47].

In high-parameter panels, the limited availability of antibodies conjugated to diverse fluorochromes often forces trade-offs in design. Understanding and managing spectral overlap is essential for a clear marker resolution. For example, Futamura et al. (2015) highlighted challenges with spectrally adjacent fluorochromes such as AcGFP and FITC, where high intensity ratios complicate signal separation [48]. Similarly, one-way spillover between pairs like APC and APC-Cy7 can produce elongated, elliptical data distributions, further complicating analysis. To mitigate these issues, spectral unmixing algorithms have been developed to leverage unique spectral profiles for an enhanced wavelength resolution and more accurate analysis, particularly in immunology and cancer research [49].

After selecting fluorochromes, assigning them to markers is crucial—brighter fluorochromes should be matched with low-expression markers to maximize the resolution and based on co-expression. Incorporating metrics like the stain index reduction or spillover spreading value can allow for assessing the potential data distortion. Additionally, considering co-expression patterns during panel design minimizes spillover among frequently co-expressed markers, ensuring optimal data quality. A detailed marker-by-marker review, leveraging tools such as the single stain index (SSI) equation, followed by antibody titration, helps reduce non-specific binding and background noise, thereby enhancing the target signal [3].

Following fluorochrome assignment and titration, gating controls—optimized using fluorescence-minus-one (FMO) or fluorescence-minus-multiple (FMM/FM-X) controls—are essential to account for signal spread. Spectral reference controls (SRCs) further aid in reliable unmixing and evaluating the impact of signal spread and fluorochrome overlap [14]. If problems arise, adjustments such as modifying antibody concentrations, sequential reagent addition, or reassigning fluorochromes can help mitigate false positives and negatives, ensuring robust data across both spectral and conventional flow cytometry platforms. Ultimately, each marker requires a unique fluorochrome with minimal overlap, a principle that underpins effective panel design, as highlighted by Park LM et al. (2024) [30,50].

**Figure 3:**
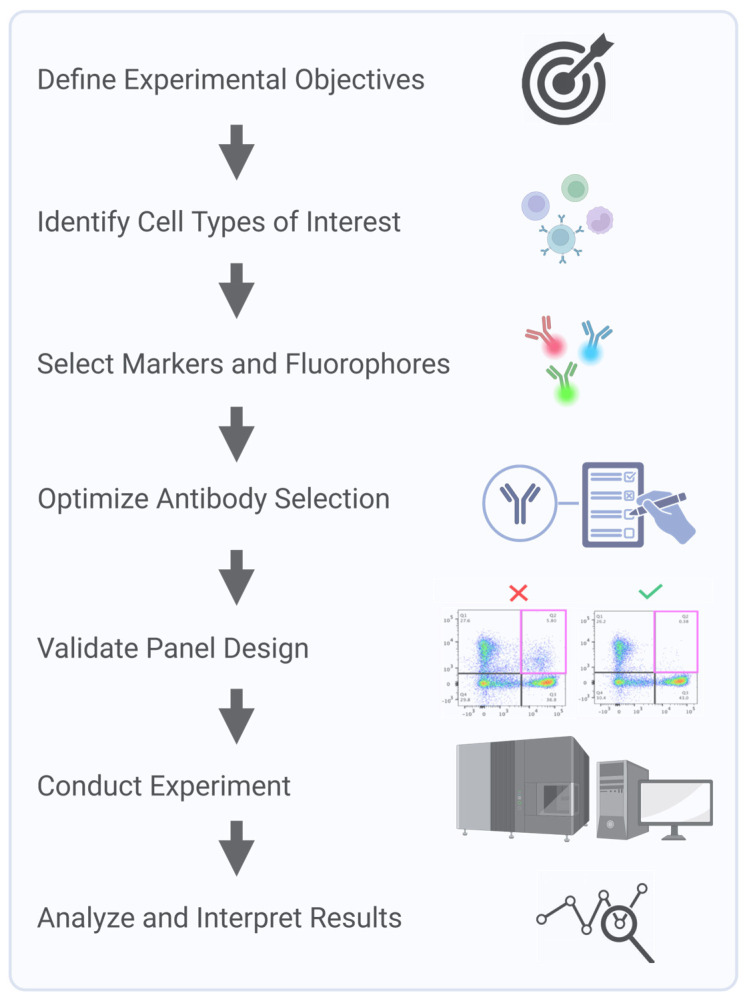
Key steps in multicolor panel design and assay optimization for spectral cytometry.

Clinical flow panels for spectral cytometry can incorporate up to dozens of markers. Successful design of these high-dimensional panels requires iterative testing and validation to account for inter-individual variability in marker expression and to ensure a consistent performance across patient populations [22]. Furthermore, the investigator must understand the biology of the samples being interrogated. For example, in leukemia, expression levels and co-expression patterns of markers can be observed that would never be encountered in normal blood cells. Consequently, during panel design and validation steps, clinically relevant samples representative of the range, and a combination of marker expressions that will be encountered, must be used. Panel design using only healthy donor samples will not recapitulate potential problems arising with ectopic or a co-expression of markers not seen in the normal population. Key steps in multicolor panel design and assay optimization for SCF accounts start with a process of defining the experimental question and identifying critical metrics, such as fluorescence intensity, marker frequency, or cell counts, to ensure the design focuses on collecting the most relevant data. Next, markers are carefully selected to accurately define the cell populations of interest, with details recorded in a cell identification table that includes key populations, markers, and co-expressions. Due to variability in marker expression across individuals and disease states, validation is critical to ensure a reliable detection across diverse samples. Key considerations, including target density, fluorochrome brightness, and antibody optimization, are integral to creating a successful and reproducible panel (Figure 3).

### 3.4. Panel Optimization for Clinical Use

Optimizing multi-parameter panels for clinical use presents some challenges. In addition to basic challenges discussed in the preceding section, clinical assays must also account for operational and biological variability, the need for iterative testing, and the requirement for platform and assay standardization across laboratories.

Rigorous validation of unmixing algorithms is essential for ensuring reliability and reproducibility in SFC, not only in clinical applications, where accurate biomarker identification is crucial, but also in any context requiring standardized results across multiple sites or studies [51]. Such validation typically involves cross-referencing unmixing outputs with known reference standards and evaluating algorithm performance under diverse experimental conditions to ensure consistent accuracy [20,52,53]. In parallel, ongoing advances in fluorochrome panel design and SFC instrumentation aim to minimize spectral overlap, thereby reducing unmixing complexity and improving overall analytical reliability [54]. Furthermore, integrating machine learning techniques into unmixing workflows is emerging as a promising strategy to further enhance accuracy in complex, high-dimensional analyses [55,56]. Ultimately, effectively resolving spectral overlap and continuously refining unmixing methods are critical for achieving reproducible SFC results across laboratories, enabling consistent multi-site studies and trials, and thereby advancing the technology’s broader applicability in both research and clinical domains.

### 3.5. Spectral Flow Cytometry Unmixing Is Enhanced by AI and Machine Learning

Machine learning (ML) and AI are addressing key challenges in SFC, including spectral overlap, noise, and variability across experiments. Traditional compensation methods often fail in high-dimensional panels, introducing artifacts and obscuring rare populations [57]. Recently, a variety of computational tools and algorithms have been developed to improve spectral unmixing and data quality [20,58,59]. AutoSpill is an algorithm that automates spillover matrix calculation using robust regression, and autofluorescence extraction simplifies the analysis of multicolor flow data and has been demonstrated to reduce iterative manual adjustments [57], while blind unmixing approaches such as NMF-RI bypass the need for reference controls by employing non-negative matrix factorization rank initialization [59]. This method can unmix highly complex, overlapping spectra without user-provided reference spectra, although it cannot be used in every scenario. Statistical models like Poisson regression improves accuracy for low-signal populations by explicitly modeling photon-counting noise. By treating the signal as a Poisson process, this approach accounts for the fact that measurement variance increases with signal intensity [1]. AI methods such as genetic algorithms and autoencoders explore the high-dimensional parameter space to optimize unmixing. For example, genetic algorithms can iteratively evolve a population of unmixing solutions toward an optimal solution, and autoencoder neural networks can learn complex non-linear unmixing transformations [58,60]. Several supporting platforms have been developed to improve data quality prior to or during unmixing. FlowAI (FlowAI v1.8.4, https://bioconductor.org/packages/flowAI, accessed on 14 June 2025) detects and removes anomalous events from flow data [61] to ensure clean inputs. CytoNorm (v1.1.0, https://github.com/saeyslab/CytoNorm, accessed on 14 June 2025) performs batch effect normalization [62] to make fluorescence intensity distributions comparable across runs. Spectracular (Spectracular v1.0, https://biosurf.org/spectracular, accessed on 14 June 2025) is an open-source tool that assists in optimized panel design and unmixing analysis [63]. These advances improve unmixing accuracy, support reproducible high-dimensional analysis, and facilitate reliable diagnostics across clinical sites [22,63,64].

### 3.6. Biological Variability

Genetic, environmental, and physiological factors can impact marker expression in biological samples such as blood and tissue, resulting in significant variability across individuals [65,66]. For example, in studies involving immune cell profiling, Odak et al. (2022) and Edwards et al. (2023) have found substantial variability in the expression of surface markers like CD4 and CD8 among healthy individuals, which can complicate the interpretation of immune responses [12,21]. Even within the same individual, different cell populations can express markers at varying levels. Biological samples are subject to dynamic changes due to factors such as disease progression, treatment responses, and circadian rhythms. These fluctuations can lead to inconsistent results when measuring marker expression over time [6,67]. For example, the expression of activation markers on T cells may vary during different phases of an immune response [21,68].

This intra-individual variability can be particularly pronounced in conditions such as cancer, where tumor microenvironments can influence the expression of immune checkpoint markers [69]. For instance, the expression of programmed cell death protein 1 (PD-1/CD279) and check-point blockade of cytotoxic T lymphocyte antigen 4 (CTLA-4/CD152) may differ among tumor-infiltrating lymphocytes compared to circulating T cells [70]. Intra-individual variability can be reflected in metabolic profiles. In the study of Heieis, G.A., et al., the authors demonstrate the impact of tissue-specific environments on macrophage metabolic profiles and adaptation [71]. They reveal distinct metabolic phenotypes in macrophages across various tissues, where liver macrophages (Kupffer cells) display high levels of GLUT1, PKM, and CD98, while alveolar macrophages in lipid-rich environments exhibit high CD36 but low GLUT1 [71].

Biological variability cannot be eliminated, but it can be characterized and taken into account. This can be achieved by careful panel design, selecting fluorochromes, markers, and clones that have demonstrated robustness across different populations [21,30,72]. Implementing standardized protocols for sample collection, processing, and storage can reduce variability introduced by technical factors. For example, consistent methods for isolating peripheral blood mononuclear cells (PBMCs) help to minimize variations due to processing differences, or establishment of robust pipelines for high-throughput sample processing help reduce technical variability between batches [18,73]. Jensen and Wnek (2021) explored how different sample types—whole blood, fresh PBMCs, and cryopreserved PBMCs—affect the variability of immune cell populations using a standardized immunophenotyping panel designed for spectral cytometry [14]. Their 25-marker panel, developed for the Cytek Aurora platform, was designed to identify a wide range of immune cell types (T cells, B cells, NK cells, monocytes, dendritic cells, myeloid-derived suppressor cells (MDSCs), etc.). The study evaluated how consistent the panel’s results were across these sample types and found that over 95% of the immune populations analyzed showed high precision, with variability under 20% for repeated measurements. However, certain markers (e.g., CXCR3 on memory CD4 T cells), were highly sensitive to storage conditions. This population increased dramatically—several fold—when blood samples were stored for 24 h before processing, highlighting the importance of storage conditions. Similarly, PBMC isolation and cryopreservation caused noticeable changes in some markers and rare cell populations compared to donor-matched fresh blood samples. Specifically, significant changes were observed in the ratio of CD14hiCD16lo/CD14loCD16hi monocytes, the CD4/CD8 T cell ratio, a shift from naïve CD45RA + regulatory T cells to memory CD45RA−Tregs, disruption of CD38 expression on CD4 T cells, and increased M-MDSCs and Th1 cells. This research emphasizes the reliability of the panel for immune monitoring in clinical studies while underscoring the need to carefully manage sample handling and storage to ensure accurate results [14].

Biological variability poses key challenges in spectral flow cytometry, especially when applying panels optimized on healthy donors to disease samples like chronic lymphocytic leukemia (CLL). CLL samples typically contain extremely high B-cell frequencies and altered antigen expression, such as dim CD20, CD22, and Ig light chains, and aberrant CD5 expression on B cells [74,75]. These deviations can impair marker resolution, particularly when gating strategies rely on internal negative populations that may be absent.

High B-cell counts can also saturate antibody reagents or detectors, reducing quantitative reliability. If antibodies optimized for healthy samples are underdosed, target sites may be incompletely stained; if overdosed, detector saturation and spillover may occur, particularly with bright fluorochromes [76]. Reagent titrations must account for these shifts, and optimal fluorochrome pairing (e.g., bright dyes for dim markers) is essential [77].

Autofluorescence and unmixing accuracy can vary due to shifts in cellular composition. Spectral panels may require adjusted controls or unmixing algorithms when applied to CLL-dominant samples. Dim marker detection also suffers if panel assumptions about antigen brightness do not hold, as seen in clonal light chain restriction or dim CD20 staining [75].

Panel misinterpretation is a clinical risk. For example, a follicular lymphoma case was initially misclassified as CLL due to overlapping marker expression [78]. The EuroFlow consortium (Groningen, The Netherlands) recommends validating panels on both healthy and malignant samples to avoid such pitfalls [79].

### 3.7. Examples of Complex Panels and Their Clinical Applications

A complex SFC panel used for deep immunophenotyping in hematological malignancies such as acute lymphoblastic leukemia (ALL) or acute myeloblastic leukemia (AML), that includes markers for various cell surface proteins, differentiation antigens, and intracellular signaling molecules, allows for the comprehensive analysis of different leukocyte subsets, including rare populations that may indicate MRD [22,23]. The complexity of these panels lies not only in the large number of markers but also in the need to accurately detect and quantify these rare populations against a background of normal cells [2,22,23,63,80,81]. Another example is the use of SFC panels in immune monitoring during cancer immunotherapy. These panels often include markers for T-cell activation, exhaustion, and memory status, as well as markers for other immune cell types such as natural killer (NK) cells and dendritic cells [82]. The goal is to monitor the immune response to therapy and identify biomarkers that can predict treatment outcomes. The complexity here arises from the need to track multiple immune cell subsets simultaneously while ensuring that the panel is sensitive enough to detect subtle changes in marker expression that may be clinically significant.

The high-dimensional capabilities of SFC offer significant advantages over CFC in detecting rare leukemic phenotypes that could have diagnostic or prognostic significance. For example, SFC can simultaneously track co-expression patterns across 20+ markers, enabling the identification of aberrant combinations that would be impossible to detect with conventional 8–10-color panels. This is particularly valuable in cases where leukemic blasts display only subtle deviations from normal differentiation pathways or express unusual marker combinations. Even when leukemic cells comprise < 0.01% of the total population, SFC’s enhanced resolution can distinguish these cells based on their unique spectral signatures [2,22,23,80]. This capability could potentially improve MRD detection thresholds and reveal previously uncharacterized leukemic stem cell populations with distinct treatment response profiles [48,81]. While continued research is needed to fully document specific immunophenotypes that are exclusively detectable by SFC, the technology’s ability to capture the full complexity of the leukemic landscape represents a significant advancement in hematological diagnostics.

To address the challenges of panel complexity in SFC, future efforts should focus on developing more sophisticated dyes, advanced software tools for spectral unmixing, and standardized panel design practices. While many traditional fluorochromes used in CFC—such as FITC, PE, and APC—are also compatible with SFC, several proprietary dye families have been developed to exploit the unique capabilities of SFC and overcome key limitations of legacy dyes. Proprietary fluorochromes such as BD RealYellow™ and RealBlue™ (BD Biosciences, San Jose, CA, USA), BioLegend Spark™ and Fire™ (BioLegend, San Diego, CA, USA), Bio-Rad StarBright™ (Bio-Rad Laboratories, Hercules, CA, USA), Thermo Fisher NovaFluor™ (Thermo Fisher Scientific, Waltham, MA, USA), and Biotium CF^®^ (Biotium, Inc., Fremont, CA, USA) dyes offer enhanced brightness, reduced spillover, minimal cross-laser excitation, and spectral uniqueness, all of which improve resolution and enable higher-dimensional panel design [83,84,85,86]. For example, RealYellow 586 matches the brightness of PE but with significantly less cross-laser excitation, reducing background in blue-laser channels and allowing PE and RY586 to be used together—an advantage unique to SFC [84]. NovaFluor dyes offer narrow, clean emission profiles and low spectral spread, which lowers error propagation during unmixing and improves the separation of dim populations [85]. BioLegend’s Fire™ tandems, like APC/Fire 810, exploit far-red and near-infrared ranges largely unused in CFC, thus expanding the number of usable parameters in a single panel [83]. StarBright dyes, based on nanoparticle technology, provide exceptional brightness and spectral distinctiveness but are not yet validated for intracellular staining (Bio-Rad, 2023). Meanwhile, Biotium’s CF^®^ dyes, PEGylated for high solubility and low non-specific binding, contribute to cleaner staining profiles in complex tissues [87]. These dyes also address CFC limitations like tandem dye instability, autofluorescence sensitivity, and limited laser channel utilization. Nevertheless, proprietary dyes may require additional considerations—such as Fc-blocking buffers or spectral reference updates—and availability for some targets remains limited. Overall, their engineered spectral and chemical properties offer tangible advantages in high-parameter SFC, supporting more flexible and accurate panel design than conventional fluorochromes allow [86,88].

Automated panel design algorithms have been developed to support researchers in creating optimized panels for SFC. For example, EasyPanel (Version 2.0) is an intelligent tool that suggests panel configurations based on user-defined inputs, helping to streamline the design process for both traditional and spectral cytometers (flow-cytometry.net). Similarly, Cytek^®^ Biosciences (Cytek Biosciences; Fremont, CA, USA) offers the SpectroPanel tool (Version 1.0), which automates fluorochrome-marker assignments (based on reagent brightness, emission profile, and spread, as well as antigen density and co-expression patterns), reducing the manual effort required to initiate panel development (cytekbio.com). While these tools can facilitate initial design steps and manage the complexity of high-parameter SFC, it is important to note that their effectiveness depends on underlying assumptions. Many current algorithms do not fully account for critical biological variables such as marker co-expression, antigen density, or expression modulation following treatment. Therefore, expert review and empirical validation remain essential to ensure robust and biologically relevant panel performance.

New dyes like Real Yellow™ and Real Blue™, APC/Fire™, PE/Fire™, and PerCP/Fire™ have unique emission profiles which enable greater flexibility in panel design, when paired with widely used fluorochromes. Spark Dyes provide reliable results with their narrow profiles and resistance to quenching from fixation buffers. Bio-Rad’s StarBright Dyes excel with unique spectral signatures and the absence of cross-laser excitation, delivering exceptional brightness for resolving rare populations and low-density antigens. Biotium’s CF^®^ dyes and Cytek cFluor dyes address issues such as non-specific binding and aggregation through pegylation, ensuring consistent performance across visible, far-red, and near-IR spectra. NovaFluors™, developed with the Phiton™ platform, (Thermo Fisher Scientific., Waltham, MA, USA) eliminates cross-laser excitation, offers clean emission profiles to reduce signal spread, and features digital brightness for precise tuning to antigen density, supporting deeper phenotyping and panel expansion. It is also important to highlight potential limitations of some of these fluorochromes, including tandem stability, as shown by Park et al. for PE/Fire™ 810 and PerCP-eFluor™ 710 [30]. The summary of fluorochrome families for SFC applications is presented in Table 2. Issues with Novafluor performance have also been reported [30].

In addition to dye development, a distinct category of advanced analytical software tools incorporating machine learning algorithms is essential for managing high-parameter panels. Unlike the previously mentioned panel design tools that focus on initial fluorochrome-marker assignments, these more sophisticated analytical platforms address the downstream challenges of complex spectral data. These advanced tools can predict spectral overlap with greater precision than traditional compensation matrices, dynamically optimize fluorochrome selection based on instrument-specific configurations, and provide real-time assistance for panel troubleshooting and refinement. Examples include SpectroFlo^®^ software (SpectroFlo^®^ version 2.2) with its automated unmixing algorithms, FlowJo™ (FlowJo, LLC., Ashland, OR, USA) Spectral Unmixing Wizard for post-acquisition analysis, and emerging AI-based platforms that can adapt to instrument-specific variations and predict optimal panel configurations based on continuously updated spectral libraries. 

Standardization efforts should also be prioritized, including the creation of universal guidelines and reference panels that enable cross-laboratory comparisons and benchmarking. Table 3 provides an updated overview (as of January 2025) of published optimized multicolor immunophenotyping (OMIP) panels that employ spectral flow cytometry for human cellular subset analysis (Table 3). Collaboration between academic researchers and industry partners will be key to accelerating the development, validation, and implementation of complex panels. By leveraging the strengths of both sectors, these partnerships can address the technical and logistical challenges of panel complexity, ensuring the continued advancement of spectral cytometry.

### 3.8. Validation of Spectral Flow Cytometry Assays Is a Critical Step in Clinical Biomarker Measurement and Monitoring

The validation of SFC-based assays is fundamental, as it is for any other flow cytometry assay, to ensure the reliability and reproducibility of data, particularly in clinical applications where biomarker detection plays a pivotal role in diagnosis, prognosis, and therapeutic monitoring. Assays must be validated to confirm they measure the intended analytes with accuracy, precision (repeatability and reproducibility), sensitivity (limit of detection), and specificity, to ensure that any observed variation can be attributed to biology, as outlined by the International Conference on Harmonization (ICH) guidelines [34]. This validation process becomes even more stringent when translated into regulatory settings, where compliance with guidelines from agencies such as the U.S. FDA or EMA is required. These regulatory frameworks mandate robust validation to prevent false positives or negatives, which could have direct clinical consequences. However, to date, the CAP flow cytometry checklist is not updated for spectral cytometry [7].

Only after the assay is properly developed should the validation process begin. The complexity of the validation protocol depends on several factors, which include the number of instruments, laboratories, operators, and the context of use (COU) of the assay. These factors will determine which parameters, such as precision (repeatability and reproducibility—intermediate precision, inter-operator and inter-instrument variability), detection capability (limit of blank—LoB and lower limit of quantification—LLoQ), stability, and carry-over, should be included for evaluation [35].

Advanced computational approaches are also being integrated into validation pipelines to account for the high-dimensional data generated by spectral cytometry [20,30,73,77].

In clinical settings, validated assays are crucial for producing reliable data that guide patient management. Without thorough validation, clinicians cannot confidently interpret complex datasets, risking misclassification of cell populations, misdiagnosis, or making inappropriate treatment decisions [91]. Recently, Jensen and Wnek (2021) outlined the validation of a 25-marker spectral cytometry immune monitoring assay, incorporating key steps to ensure its reliability and reproducibility [14]. Their process included antibody validation to confirm specificity, sensitivity, and cross-reactivity, ensuring antibodies’ performance in the assay; standardized sample preparation protocols to minimize variability in blood collection, PBMC isolation, and cryopreservation; and data analysis, where a standardized hierarchical gating strategy was developed within the study parameters. While traditional manual gating approaches (including hierarchical strategies) are inherently subject to operator variability, the authors implemented specific controls like gating templates and consensus review to maintain consistency across operators. Importantly, they also compared these manual approaches with FlowSOM (Bioconductor (R package), typically v1.24.0) analysis (an automated clustering tool) to validate the complementary strengths of both methods [92]. This comparison highlights the emerging best practice of leveraging both human expertise and computational approaches for high-dimensional data analysis. Manual gating provided interpretable biological insights while automated methods offered enhanced objectivity and reproducibility, particularly valuable for the complex spectral signatures in high-parameter cytometry. They assessed intra-assay precision by analyzing healthy donor samples in replicates, calculating % coefficient of variation (CV) for measurement consistency. Stability testing evaluated sample durability through blood storage, PBMC isolation, and cryopreservation to maintain population consistency. Finally, performance characterization assessed analytical robustness. In their validation, the authors found that over 95% of gated populations showed high precision across experimental replicates. However, the phenotypic E-MDSC (myeloid-derived suppressor cell) population did not meet this standard, as it exhibited poor repeatability in blood samples, with a %CV exceeding 20%, or was unmeasurable in PBMCs due to a cell count below 100 with the acquisition settings used. After PBMC isolation, 42 out of 49 gated immune cell populations (86% after 24 h) remained stable, indicating minimal changes in cell numbers for most populations. Plasmacytoid dendritic cells (pDCs) and CD15 + CD11b + low-density granulocytes (LDGs) exhibited statistically significant differences in cell counts between blood samples and isolated PBMCs, suggesting some variability in these subsets due to isolation. In terms of cryopreservation, the authors reported that 36 out of 49 gated populations (73%) did not exhibit significant changes after storage at −80 °C for 7 or more days. However, there were notable changes in specific populations, including shifts in the CD4/CD8 ratio and an increase in M-MDSCs.

Given the increasing importance of cytometry in personalized medicine, it is necessary to adhere to guidelines (CLSI H62) for a more standardized approach to flow cytometry assay validation across clinical laboratories globally [93]. Consideration must also be given to expanding current guidelines, creating requirements for proper reference controls, and establishing reference testing programs, as well as tools and workflows for fast and robust data analysis. As spectral cytometry continues to evolve, the integration of AI/ML approaches with traditional validation methods will likely become standard practice, offering solutions to the inherent limitations of manual analysis while preserving the biological insights that come from expert interpretation.

### 3.9. The Critical Role of Standardization in Spectral Clinical Flow Cytometry: Addressing Variability Across Laboratories

Standardization of instrument setup, laboratory procedures, and analysis protocols is essential when a flow cytometry assay is repeatedly employed, especially for clinical applications, such as immunophenotyping or cancer diagnostics, where incorrect results, due to technical variability, can have deleterious implications for patient care.

Performance reproducibility and the ability to compare results over time and across different laboratories are crucial in clinical trials and assessed during validation. To ensure success in passing validation criteria, when introducing a new instrument in the lab, installation should start with initial characterization to establish the baseline performance, to enable inter-instrument/inter-site comparisons. Tools like stain index reduction (SIR), the cross-stain index (CSI), and the spillover spread matrix (SSM) identify performance issues and optimize panel design by highlighting the marker resolution and spillover. Long-term tracking of CSI and SSM metrics, along with advanced QC and NIST-traceable standards, ensures a consistent performance and reliable data, enhancing the quantitative capabilities of spectral cytometry in complex panel analysis [51]. Flow cytometry settings can vary across laboratories, with differences in instrument configurations, target values, and calibration practices. These inconsistencies can lead to divergent data, even when analyzing identical sample types or cell populations. To ensure meaningful comparisons across instruments—especially those with different optical configurations—fluorochrome-specific standardization is essential. Unlike hard-dyed calibration beads, which do not fully reflect the performance characteristics of individual fluorochromes, fluorochrome-specific controls help account for differences in detector sensitivity and spectral overlap. Even among identical instruments, such standardization enhances data reliability and reproducibility [91]. During instrument daily QC, fluorescent beads are used, based on the manufacturer’s recommendations, to assess instrument performance and monitor cytometer stability across time. For the optimal marker resolution in each assay, the acquisition settings need to be optimized to find the detector gain or voltage values that ensure the highest signal-to-noise separation. This universal instrument setup standardization is not just a technical goal but a clinical necessity.

Sony and Cytek Biosciences have developed distinct approaches to instrument setup standardization to ensure data consistency and reproducibility across different days, instruments, and sites. For example, Sony incorporates a standardization mode that calibrates each detector channel to an optimized master specification, normalizing scatter and fluorescence sensitivity across instruments of the same model. This global specification refers to a master reference configuration to which each detector voltage is adjusted for matching fluorescence intensities across units. During daily QC, the system recalculates voltage scaling factors to restore alignment to the global standard, compensating for detector drift and ensuring consistent target values across lasers and detectors. Cytek Biosciences offers cytometers equipped with a similar feature designed to reduce instrument-to-instrument variability, thereby enhancing data reproducibility. Their instruments use acquisition settings optimized using biological human samples to find the sweet spot for a high resolution, minimal spread, and preserved spectral signatures. These optimized settings are named CytekAssaySetting (CAS) and define the target fluorescence intensity values for each detector. If the intensities deviate from the target during daily QC, detector gains are automatically adjusted to match, compensating for detector drift and inconsistencies in target values. This approach maintains the resolution and signal stability over time and supports harmonization across instruments, including sorters and analyzers, as shown by Park et al. (2024) [30]. These features minimize signal variability within and between instruments, yielding consistent fluorescence intensity measurements and highly reproducible results in longitudinal studies and multi-site collaborations. By normalizing detector gains or voltages to achieve uniform target intensity values, the standardization also enables the reuse of single-color reference controls from a spectral library without the need for fresh controls in each new experiment. BD’s Cytometer Setup and Tracking (CS&T) system establishes a baseline instrument performance using multi-level fluorescent calibration beads. It records median fluorescence targets for dim, mid, and bright beads during the initial setup and adjusts detector voltages daily to restore these values. This ensures that the system’s sensitivity and compensation parameters remain stable over time. However, voltration might be needed to define the optimal acquisition settings for a specific sample or panel to achieve the highest data resolution.

Developing and implementing SOP for instrument calibration, sample preparation, acquisition, unmixing adjustment, and data analysis can help minimize variability. SOPs should be detailed and specific, ensuring that all laboratories follow the same protocols. This approach has been advocated in the literature to enhance reproducibility [94]. Incorporating rigorous quality control (QC) measures, such as the use of standardized reference samples and regular calibration checks of instruments, can ensure a consistent performance. Regular QC checks can help identify and rectify discrepancies in instrument settings or sample preparation methods [95,96]. Discrepancies in sample handling, such as the method of cell staining or the timing of analyses post-staining, can lead to variability in results. A review by Mair and Tyznik (2019) highlighted that inconsistent staining protocols could affect the fluorescence intensity and, consequently, the accuracy of marker detection in multi-parameter panels [97]. Different laboratories may employ distinct algorithms or thresholds for data analysis, leading to variations in how results are interpreted [97]. For example, a study by Fan, et al., 2022 found that using different unmixing algorithms resulted in markedly different estimates of rare cell populations in hematological malignancies [58].

Conducting inter-laboratory studies can facilitate the identification of sources of variability and help refine standardization efforts. Collaborative studies where multiple laboratories analyze the same samples using standardized acquisition settings can provide valuable insights into differences and promote uniformity [98]. Implementing training programs for personnel involved in flow cytometry can ensure that staff are knowledgeable about standardized practices. Certification programs may help maintain consistency in how assays are performed and analyzed across different sites [99]. Creating universal multi-parameter panels that are optimized for use across different instruments and laboratories can help streamline workflows and reduce variability. Such panels would be validated across multiple platforms to ensure consistency in results, regardless of where the testing is performed. To date, few clinical trials are completed using spectral flow; however, the adoption of the technology in this space is increasing every day, making standardization ever more important [100,101].

### 3.10. Efforts and Challenges in Achieving Consistent Results

The development of universal protocols and calibration standards has been a priority in the field. Several organizations, including the International Society for Advancement of Cytometry (ISAC), have been actively involved in establishing guidelines that laboratories can adopt to minimize variability. These efforts include the creation of reference materials and calibration beads that allow laboratories to ensure their instruments are functioning correctly and consistently over time.

### 3.11. Role of International Standards and Guidelines

International guidelines play a crucial role in harmonizing practices across different laboratories. Organizations such as the CLSI and the World Health Organization (WHO) are working toward establishing universal standards for flow cytometry that can be applied globally. Efforts to standardize flow cytometry across research and clinical settings have been driven by several key consortia and projects, each addressing unique challenges in reproducibility and data harmonization. These groups have focused on areas such as MRD detection, immune profiling, and interlaboratory variability. The table below summarizes the major groups involved in standardization, highlighting their specific focus areas and the contributions they have made to advance the field. By developing comprehensive protocols, uniform reagent panels, and automated tools, these initiatives have significantly improved the consistency and reliability of flow cytometry applications across diverse settings [101] (Table 4). These guidelines are critical for ensuring that data are comparable across clinical studies, thereby facilitating the broader adoption of this technology in research and diagnostics.

One promising area of focus is the implementation of standardized instrument calibration procedures, which ensure that data collected on different instruments is comparable. For instance, calibration beads with known fluorescence intensities are increasingly being used to standardize fluorescence measurements across different cytometers.

Additionally, the introduction of proficiency testing programs allows laboratories to evaluate their performance relative to standardized benchmarks, promoting continuous improvement and adherence to best practices. Programs like CAP provide standardized wet and dry challenges. Wet challenges use cell lines, either undiluted or diluted in preserved whole blood, to mimic clinical samples. Dry challenges include case summaries, clinical data, and flow cytometry plots for interpretation [102]. External quality assessment programs such as UK NEQAS for Leucocyte Immunophenotyping (UKNEQAS LI, External Quality Assessment Program for Flow Cytometry. https://www.ukneqasli.co.uk/ (accessed on 14 June 2025)). are also instrumental in standardization efforts. However, the path to universal adoption of these guidelines is not without obstacles. Laboratories may lack the resources or expertise to implement complex calibration standards or/and may resist changes to established workflows. That being said, standardization is essential for improving data reliability and reproducibility across laboratories. International guidelines and standards will play an increasingly important role in this process, but ensuring consistent implementation across different laboratories requires further collaboration, technological innovation, and regulatory oversight.

### 3.12. Reference/Quality Controls in Spectral Flow Cytometry: Critical for Data Integrity and Reproducibility

Proper controls in spectral flow cytometry are essential at multiple levels to ensure data quality, reproducibility, and accurate interpretation. Broadly, these controls fall into three categories: (1) single-color reference controls for spectral unmixing, (2) daily instrument quality control (QC) and standardization measures, and (3) experiment-specific or panel-level controls (e.g., FMO tubes, positive/negative sample controls). In a spectral flow cytometry experiment, all three types of controls work in tandem to verify that the instrument is performing optimally, that fluorescence spectra are unmixed correctly, and that the biological results are trustworthy. Setting up single-color reference materials (SCRMs) is widely recognized as one of the most labor-intensive aspects of SFC, particularly when establishing new panels with many fluorochromes. Unlike CFC, where fewer compensation controls are needed, SFC often requires 20–40+ well-matched SCRMs, each capturing a fluorochrome’s full emission spectrum for accurate unmixing—a process that is sensitive to changes in reagent lot, cell type, and instrument conditions [7]. To reduce this burden, spectral cytometry platforms such as Sony and Cytek allow users to store and reuse spectral signatures in reference libraries, enabling a faster setup for recurring panels [88]. However, these libraries have limitations: they require regular validation, may not apply across lot changes or cell types (especially with tandem dyes), and are not suitable for novel fluorochromes without freshly acquired reference data. Thus, while libraries can streamline routine workflows, new or evolving panels still demand fresh SCRMs for accurate spectral unmixing [7]. Below, we discuss each category, highlighting their distinct roles and best practices, and we summarize them in Table 5.

Spectral flow cytometry requires reference controls (single-color controls) for each fluorochrome in the panel to accurately unmix overlapping emission spectra [72]. These are analogous to compensation controls in conventional flow but even more critical: the unmixing algorithm uses them to define the unique emission profile of each fluorophore. Each fluorophore–antibody conjugate in the experiment must have a corresponding reference sample with that single marker positive and a clear negative population of the same material [103].

Single-Color Reference Materials: In practice, single-color reference controls can be prepared on either cells or specialized beads that bind antibodies. Antibody capture beads (e.g., BD™ CompBeads; BD Biosciences, San Jose, CA, USA or UltraComp eBeads™; Thermo Fisher Scientific, Waltham, MA, USA) are commonly used because they reliably yield a bright positive signal and a negative population in the same tube. However, an important caveat is that the spectral signature of a fluorophore on beads can sometimes differ from that on actual cells, especially for tandem dyes or fluorophores sensitive to environmental conditions. Such differences, if unrecognized, can lead to unmixing errors [72]. The best practice is to verify each single-color control on the same cell type used in the experiment whenever possible. In cases where suitable positive cells are available, a cell-derived reference may provide the most representative spectrum. Setting up single-color reference controls is one of the most labor-intensive steps in SFC. While reference libraries (e.g., Cytek’s spectral library) offer some relief, their use requires caution due to instrument-specific variability and dye stability.

Negative and Autofluorescence Controls: Each reference control must include a well-defined negative population. When using capture beads, the beads typically come in a mixture of positive and negative beads, so an unstained (autofluorescence-only) population is inherently present. With cell-based controls, one can either use a sample that contains both stained and unstained cells or mix unstained cells of the same type. A special consideration in spectral cytometry is autofluorescence—the inherent fluorescence of cells can vary significantly. Spectral unmixing systems allow explicit subtraction of autofluorescence by treating it as an additional “fluorophore” with its own reference spectrum. To enable this, an unstained sample from the same tissue or cell type is run as a reference for autofluorescence. The use of antibody capture beads is particularly helpful when markers of interest are present in a low abundance or expression is conditional, as is often the case with activation markers. However, bead performance may vary between instruments due to differences in optical configurations or unmixing algorithms [104]. It is essential to determine unmixing requirements early and maintain them throughout the study.

### 3.13. Daily Instrument Quality Control and Standardization

Daily QC ensures cytometers maintain a consistent operational state across time and locations. Flow cytometers benefit from routine QC procedures that test optical alignment, detector sensitivity, and background noise. Daily instrument QC involves running standardized particles to check that the instrument’s performance stays within established tolerances [6].

Most cytometer manufacturers provide integrated QC systems. BD’s CS&T uses fluorescent beads with known target values; Cytek’s SpectroFlo QC beads are used with CytekAssaySetting (CAS). The CAS profile is applied after each daily QC run to ensure a consistent spectral response across detectors. This allows for matching signal intensities across runs and instruments. Emerging synthetic QC tools such as FlowCytes^®^ (Slingshot Biosciences) mimic leukocyte properties and test both scatter and fluorescence in a more sample-like context.

### 3.14. Experiment-Level Controls and Panel Validation

Experiment-level controls validate the staining process and assist in data interpretation. These include fluorescence-minus-one (FMO) controls, synthetic or spiked positive controls, and reference samples.

FMO Controls: An FMO control contains all antibodies in the panel except one, helping define gating thresholds. FMOs reveal combined background fluorescence and spillover into the channel of interest. Although spectral unmixing reduces signal overlap, FMOs remain valuable for setting gates, especially in dimly expressed markers [105].

Synthetic Positive Controls and Biological Reference Samples: Synthetic controls such as TruCytes™ (Slingshot Biosciences, Emeryville, CA, USA) mimic defined cellular phenotypes, offering a consistent readout for validating assay sensitivity. Commercial biological controls like CD-Chex^®^ Plus (Streck, La Vista, NE, USA) and ClearLLab™ Control Cells (Beckman Coulter, Brea, CA, USA) provide stabilized populations for quality assurance in clinical diagnostics. These controls serve as external benchmarks and help detect reagent failures or day-to-day variability.

Advanced Data Quality Monitoring: Tools such as flowAI can detect acquisition anomalies. Other computational methods and machine learning can assist with unmixing or background correction and provide real-time quality control during acquisition [61].

Panel development and optimization require iterative testing. Tools such as cross-stain index (CSI) matrices and NxN plots help visualize resolution, spread, and marker separation. For instance, Jensen and Kim (2023) described the use of these QC tools to refine their 30-color iCoreDrop panel through multiple fluorochrome reassignments and resolution optimization [106].

**Figure 4:**
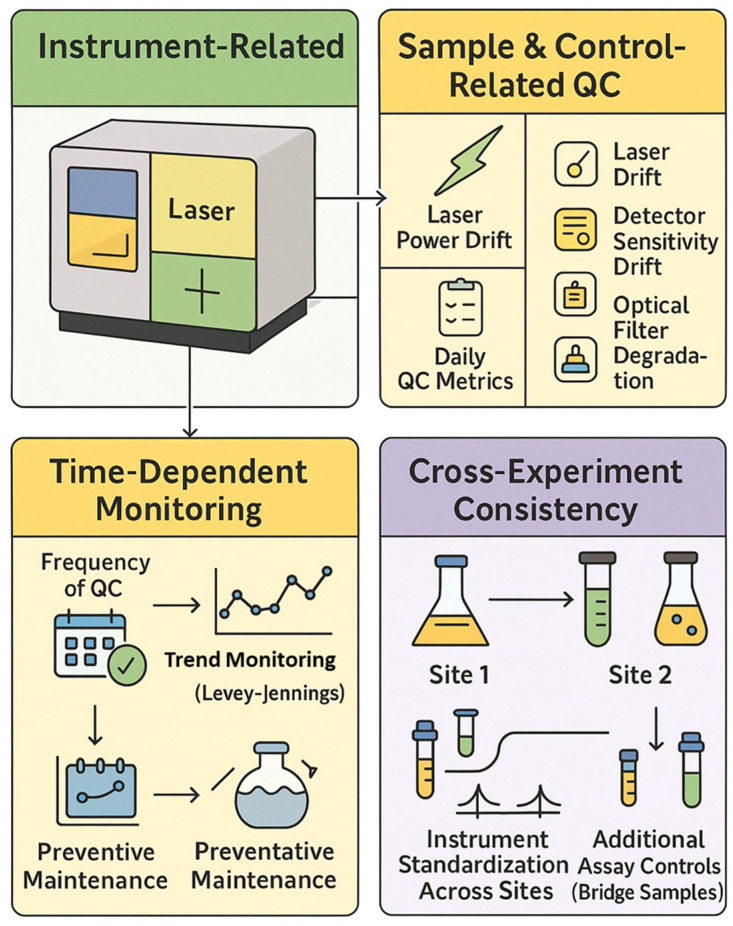
Quality control framework in spectral cytometry.

Rigorous application of reference controls, daily instrument QC, and experiment-level controls are essential for generating reproducible, high-quality data in spectral flow cytometry (Figure 4).

### 3.15. Spectral Data Files and Unmixing Models

Spectral unmixing in flow cytometry offers a sophisticated yet not fully standardized approach to quantifying fluorochrome abundance by resolving overlapping signals captured by multiple detectors [107]. Unlike traditional compensation, which adjusts for spillover between detectors, unmixing treats detector readings as mixtures of all fluorochrome signals. This enables improved separation of individual spectra compared to traditional spillover subtraction [108]. Various mathematical models can resolve mixtures of fluorochrome spectra (Table 6). Unmixing algorithms excel in managing noise, particularly noise arising from the randomness of photon emission. By using all detectors collectively, unmixing better separates noise from the true fluorescence signal [108]. However, noise correction via unmixing is not foolproof. Autofluorescence (AF) from biological samples can obscure true fluorescence and reduce signal resolution. Severe cases may impair population differentiation by introducing artifacts and false positives [4,16].

By modeling AF explicitly, unmixing provides more reliable and accurate fluorochrome quantification, particularly in complex biological samples where AF is prevalent. This results in more robust data interpretation, essential for high-precision experiments [1]. Table 7 compares the autofluorescence handling strategies implemented in leading spectral cytometry instruments and analysis platforms. 

From a biological standpoint, the method chosen for spectral unmixing has far-reaching consequences for data fidelity, especially when analyzing subtle marker expression or rare immune subsets. Ordinary Least Squares (OLS), while computationally efficient and widely used, has limitations in its baseline implementation—most notably, it can produce unphysical negative values and exhibit signal spread that compromises the resolution of dim markers and subset boundaries. However, as shown by Spasic et al. (2024), these limitations can be mitigated through enhanced spectral modeling, such as the inclusion of autofluorescence endmembers in the mixing matrix [20]. Their study demonstrates that even with OLS, careful construction of the unmixing matrix significantly improves the resolution, particularly for low-abundance markers, without requiring a change in the underlying algorithm. Non-Negative Least Squares (NNLS) resolves negatives but may artificially zero out weak true signals [4]. Weighted Least Squares (WLS) improves on both by accounting for channel-specific noise, preserving dim marker distinctions without introducing negatives [1,110]. However, Poisson regression clearly outperforms all others, offering the most biologically accurate representation by modeling photon noise directly. It is uniquely capable of preserving dim marker fidelity (e.g., CD25, PD-1) (4), enabling accurate immune subset identification (e.g., memory B cells, CD56 bright NK cells), and effectively handling autofluorescence as an independent spectral component. Though computationally more demanding, Poisson-based unmixing provides the highest analytical precision and consistency—essential for clinical-grade immunophenotyping, high-dimensional datasets, and translational biomarker discovery. Variance-Stabilizing Transformation (VST), while computationally efficient, functions best as an approximation and may require tuning for stability. This nuance is important for understanding the broader landscape of spectral unmixing: while standard OLS may fall short, enhanced implementations—like that used in Spasic et al. (2024) illustrate how OLS can be extended to achieve high-quality results [20]. Therefore, the earlier critique of OLS refers to its unoptimized form and not to optimized workflows. Thus, while OLS remains the underlying algorithm, the improved resolution is attributed to optimized spectral modeling rather than algorithm replacement.

## 4. Spectral Flow Cytometry’s Use in the Clinical Setting: Future Directions

### 4.1. Exploring Alternative Unmixing Algorithms

**Figure 5:**
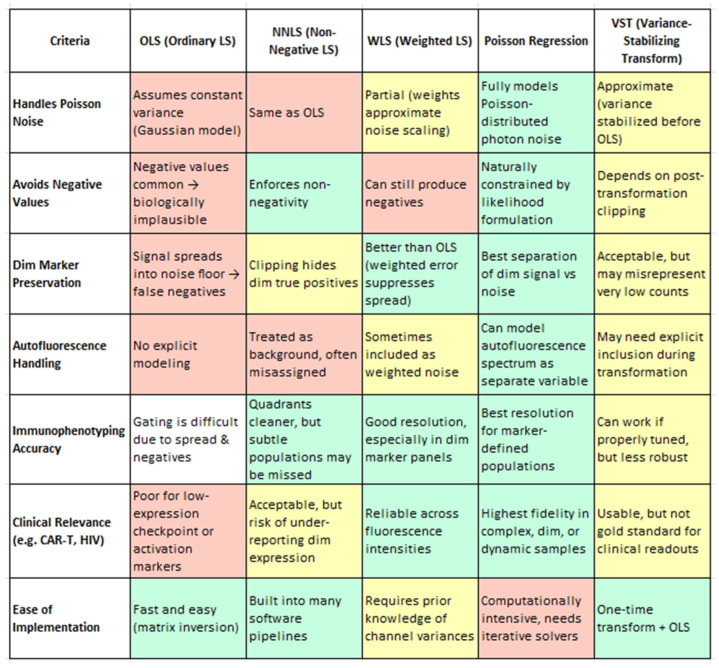
A comprehensive evaluation of five widely used spectral unmixing strategies—OLS, NNLS, WLS, Poisson Regression, and VST—assessed through the lens of biological utility in flow cytometry. The criteria include handling Poisson-distributed photon noise, preservation of dim-marker expression, mitigation of autofluorescence effects, accuracy of immunophenotyping, clinical applicability, and robustness across sample batches. Each method is scored qualitatively to reflect its practical performance in resolving biologically meaningful subsets, particularly under challenging conditions such as low signal intensities or high background noise. Color coding: Green = Recommended/Optimal, Yellow = Conditional/Intermediate, Red = Suboptimal. Criteria adapted from Novo et al. (2013), Novo, (2022) and Jameson et al. (2022) [1,16,108].

While enhanced OLS methods perform admirably, there is active interest in alternative unmixing algorithms that might further address OLS’s inherent statistical limitations. These approaches incorporate different assumptions about measurement noise and weighting of spectral channels, offering potential improvements in accuracy and reliability. Future studies should investigate and compare these techniques directly to the optimized OLS pipeline. To assist in evaluating these approaches, Figure 5 provides a qualitative comparison of five commonly used unmixing strategies—OLS, NNLS, WLS, Poisson regression, and VST—based on their biological utility in spectral flow cytometry. Evaluation criteria include their handling of photon noise, preservation of dim marker signals, mitigation of autofluorescence, robustness across samples, and suitability for clinical applications. This summary draws upon findings from Novo et al. (2013), Novo (2022), and Jameson et al. (2022) [1,16,108]. Some promising alternatives include Poisson regression-based unmixing; instead of assuming constant variance (as OLS effectively does), a Poisson unmixing algorithm models the counting noise of photons, which is more accurate for fluorescence data. In a generalized linear model framework with an identity link, the Poisson approach has been shown to yield superior unmixing results for low-intensity populations. By accounting for the fact that measurement variance grows with signal intensity (a property of shot noise), this method can improve the resolution of dim markers and reduce spurious negative values. WLS applies variable weights to different detectors based on signal intensity or noise levels. In spectral flow cytometry, brighter detectors can be given greater weight to improve fit accuracy. WLS corrects for OLS’s uniform weight limitation and may reduce the introduction of negative values and improve the resolution. Other emerging strategies include imposing non-negativity constraints, blind unmixing, and machine learning approaches. These require further validation in comparative studies.

### 4.2. Clinical Relevance and Future Validation Needs

Advances in spectral unmixing have yielded direct clinical gains, particularly in identifying rare or dim immune populations such as PD-1 low tumor-infiltrating lymphocytes and CD56 bright NK cells [1,48,81,111]. These subsets are critical for immunotherapy monitoring and precision immune profiling. Poisson-based unmixing approaches have demonstrated a superior fidelity for low-abundance signals by correctly modeling photon noise and preserving weak antigen expression [1,5,48,81,111]. However, recent work by Spasic M et al. (2024) shows that even with traditional OLS unmixing, strategic enhancements like the inclusion of autofluorescence signatures in the mixing matrix significantly improve marker resolution [20]. This highlights that algorithm optimization and the reference quality are equally critical in clinical practice.

Future studies should conduct head-to-head comparisons of Poisson regression, WLS, and optimized OLS across various clinical settings—such as MRD detection and high-autofluorescence tissue profiling. Ensuring reproducibility, robustness, and cross-platform consistency will be key for broader regulatory adoption [16]. Thus, while OLS with enhanced spectral modeling performs strongly today, alternative unmixing algorithms (Poisson, WLS) represent important avenues for continued methodological advancement.

### 4.3. Toward Standardization

A promising ongoing effort is the development of standardized, universally accepted reference materials. For instance, the international consortia aim to create biological standards that mimic human tissue environments, providing more physiologically relevant controls. The future may see automated QC systems replacing manual calibration processes, particularly as the technology advances. Standardized, non-biological reference materials could be engineered to effectively mimic the complexity of human cells, ensuring consistent and reproducible data. Achieving these developments is crucial if the life sciences community aims to establish robust, reproducible standards. Additionally, machine learning algorithms have been proposed to monitor and adjust for technical variation in real-time, offering the potential to flag errors more effectively than manual QC checks.

### 4.4. Longitudinal Studies Using Spectral Flow Cytometry

Longitudinal studies are essential for tracking disease progression, immune dynamics, and therapeutic responses. SFC, with its capacity to analyze multiple markers in a single tube, is well-suited for such studies—but technical consistency is critical. Reproducibility requires a strict adherence to assay conditions, including standardized sample handling, reagent tracking, and instrument calibration over time [14,21,73]. Establishing and routinely validating reference controls, such as cryopreserved PBMCs or batch-matched bridge samples, is necessary to detect technical drift and maintain consistency throughout the study duration [14,20,112]. Automated quality control systems and statistical models can assist in distinguishing genuine biological trends from variability due to instrument or sample-processing changes [56,73,112]. While intra-individual biological variability remains a challenge [21,66,67], SFC’s enhanced resolution makes it a valuable tool for longitudinal clinical research—provided that rigorous control strategies and harmonized protocols are in place [14,20,73].

### 4.5. Data Analysis Tools Geared Toward Spectral Flow Cytometry

The increasing complexity and dimensionality of highly parametric flow cytometry data present significant analytical challenges. Manual gating—while foundational—becomes increasingly labor-intensive and prone to user variability as panels expand beyond traditional parameters [113]. To address these limitations, a wide array of advanced computational tools has emerged, enabling more consistent, scalable, and objective data analysis. AI and ML approaches are transforming how SFC data are processed and interpreted (Table 8). These methods excel at identifying subtle biological patterns and rare cell populations in high-dimensional datasets, while reducing the reliance on manual workflows. Tools such as FlowSOM and t-SNE have gained traction for unsupervised clustering and visualization, while deep learning models are increasingly being explored to automate gating and classification tasks. A robust head-to-head comparison of clustering approaches was performed through the DREAM consortium to assess several different algorithms [114]. A novel approach to classifying cells using a non-gating combinatorics approach has also been utilized. This approach involves transforming the fluorescence values from cytometry to generate a normalized set of values and then applies functions over each cell to exaggerate bright expression in positive markers and dim expression in negative markers, allowing for easier delineation [115]. Table 8 summarizes key categories of computational tools currently used in spectral flow cytometry, along with their applications and practical considerations.

Following data cleanup for batch effects and QC, clustering and dimensionality reduction approaches such as tSNE or UMAP—commonly categorized as unsupervised due to the absence of predefined class labels—are applied. While these methods do not use outcome variables, their results are sensitive to user-defined hyperparameters, which influence the structure and interpretation. The challenge is now identifying phenotypes within clusters to identify cells of interest. Efforts by Diggins et al. have provided a sample quantitative labeling approach, known as marker enrichment modeling, which provides phenotype scores for each generated cluster. This open-source pipeline has been shown to detect enriched markers within specific clusters [126]. Furthermore, this has been built into pairwise comparison pipelines, such as the Tracking Responders Expanding (TREX) pipeline. Despite these advances, automated tools are not without limitations. Their performance is highly dependent on underlying assumptions, and most do not yet account for critical biological factors such as co-expression patterns, antigen density, or modulation of marker expression post-treatment. These nuances are especially important in clinical or treatment-responsive contexts and must be validated empirically.

Furthermore, while commercial software platforms offer comprehensive and user-friendly analysis environments, they often lack interoperability due to proprietary data structures, inconsistent formats, and high costs [107,129]. Hybrid workflows that integrate both commercial and open-source tools, particularly those with modular architectures, offer a promising path forward. These approaches can better support evolving needs in immunotherapy, infectious disease, and systems immunology—while advancing reproducibility and data harmonization across laboratories.

## 5. New Emerging Tools in Flow Cytometry and Future Potential Applications in Clinical Cytometry

The field of flow cytometry is undergoing a technological revolution with the introduction of novel tools and methodologies poised to reshape its application in both research and clinical settings. Emerging technologies like ghost flow cytometry, light-assisted scattering enhancement (LASE), and imaging cytometry have the potential to integrate seamlessly with multi-omics approaches, offering substantial advantages for patient diagnostics. As these tools transition from research laboratories into clinical practice, they promise to significantly enhance diagnostic precision, patient stratification, and personalized medicine.

### 5.1. Ghost Flow Cytometry: A Paradigm Shift?

Ghost flow cytometry, a novel technique that leverages the principles of ghost imaging, offers unprecedented sensitivity by acquiring data from photons scattered indirectly from a sample [130]. Unlike traditional cytometry, which relies on direct measurement of light scatter and fluorescence, ghost flow cytometry enables the reconstruction of high-resolution cellular images from fewer photons, potentially lowering background noise and improving signal-to-noise ratios.

Could this novel approach allow for the detection of rare cell populations with greater sensitivity than traditional flow cytometry? Early data suggest it could outperform existing techniques in detecting low-abundance markers or subtle phenotypic variations in heterogeneous cell populations [131]. However, further validation is required to determine whether the increased sensitivity and reduced sample damage associated with ghost flow cytometry can be widely applied in clinical settings, particularly in oncology, immunology, and rare disease diagnostics.

### 5.2. LASE (Light-Assisted Scattering Enhancement): Expanding the Horizon of Cellular Characterization

LASE technology, another emerging tool, uses laser-assisted scattering enhancement to improve the detection of cellular characteristics such as membrane integrity, morphology, and protein expression. By combining fluorescence detection with enhanced light scatter, LASE provides more detailed and quantitative information about the cell size, shape, and internal complexity compared to traditional forward and side scatter measurements [132]. This enhanced detection capability opens up new avenues for multi-parametric analyses, particularly in clinical diagnostics. For example, in hematological malignancies, LASE could provide a more detailed characterization of blast cells and better distinguish between different subtypes of leukemia based on subtle morphological differences. However, questions remain about how well LASE will perform across diverse clinical samples and whether its complexity may hinder its adoption in routine clinical workflows. Could this method, despite its advantages, face obstacles in terms of operational cost and ease of implementation in diagnostic laboratories?

### 5.3. Imaging Cytometry: Marrying Flow and Imaging for Enhanced Diagnostics

Imaging cytometry represents a unique convergence of flow cytometry and microscopy, allowing for the simultaneous acquisition of quantitative data and high-resolution cellular images. This hybrid approach enables the visualization of intracellular components alongside the usual fluorescence data, making it particularly powerful in applications like fluorescence in situ hybridization (FISH) [133]. FISH, used primarily for detecting chromosomal abnormalities in cancer and genetic disorders, stands to benefit greatly from the integration with imaging cytometry, enabling more a precise spatial localization of genetic probes within cells [134].

How can this approach be leveraged to improve diagnostics for conditions like leukemia, where chromosomal translocations or other genetic alterations play a pivotal role in disease progression? Imaging cytometry, when combined with FISH, may allow for earlier detection of chromosomal aberrations or mixed populations of malignant and healthy cells, potentially improving the prognosis through earlier intervention [135]. Furthermore, the ability to visualize the cellular morphology in conjunction with genetic abnormalities opens the door to more personalized therapeutic strategies.

### 5.4. Advantages of Multi-Omics Integration into Clinical Cytometry

Integrating these advanced flow cytometry tools with other omics technologies—such as genomics, transcriptomics, proteomics, and metabolomics—promises to revolutionize patient diagnosis by providing a more holistic view of disease. Multi-omics approaches are already showing great promise in oncology and immune-mediated diseases by allowing clinicians to correlate cellular phenotypes with underlying molecular mechanisms [136].

How can clinical cytometry best integrate these multi-omics techniques? One solution lies in developing standardized, interoperable platforms that can easily combine the data from flow cytometry, imaging, and omics technologies into a single, cohesive dataset. However, significant challenges remain in terms of data management, analysis, and interpretation, especially in high-dimensional datasets. Moreover, the question arises: can the healthcare infrastructure adapt quickly enough to support the integration of these complex data streams into routine clinical practice? Addressing this challenge will require robust bioinformatics tools and cross-disciplinary collaborations and solutions for data portability and current limitations in data acquisition platforms to make these technologies accessible and practical for clinical use.

### 5.5. Proposed Solutions

1. Validation and standardization: As new technologies like ghost flow cytometry, LASE, and imaging cytometry advance, establishing rigorous validation protocols will be essential to ensure their clinical applicability. Standardizing these techniques, similar to the standardization of traditional flow cytometry markers, could accelerate their adoption in clinical diagnostics.
2. Integration with multi-omics: Developing unified platforms that can handle the high-dimensional data generated by these techniques in conjunction with multi-omics data will be critical. Investments in bioinformatics infrastructure and machine learning tools will be necessary to make sense of these complex datasets.
3. Training and accessibility: Widespread adoption of these emerging technologies will require training programs to familiarize clinical staff with the new methods and their interpretation. Additionally, cost-effective models need to be developed to ensure accessibility across various clinical settings, from major research hospitals to smaller diagnostic labs.
4. Platform portability concerns in SFC: To overcome current limitations in platform and data portability within SFC, there is a clear need for the development and adoption of standardized, open data formats and interoperable spectral libraries. Leveraging the capabilities of FCS 4.0—which supports spectral matrices and metadata—manufacturers and software developers should collaborate to create universal data structures that enable the consistent interpretation of unmixing models across systems. In parallel, establishing consensus-driven calibration protocols and vendor-agnostic spectral reference databases would promote reproducibility and cross-platform compatibility. Encouraging the use of open-source analysis tools, as well as cloud-based platforms that support multiple file formats and flexible gating strategies, can further ensure that spectral data is easily shareable, interpretable, and integrable into clinical workflows. These efforts will be critical to enabling seamless multicenter studies, long-term clinical monitoring, and collaborative research across institutions using diverse instrumentation.

## 6. Conclusions

SFC as a platform is transforming clinical study analysis by enabling the interrogation of far greater markers, by analyzing the entire emission spectrum of each fluorochrome, which in turn allows for a deeper investigation of key and rare subsets when compared to CFC-deconvoluting spectral signals, which are more closely related than could be achieved with older technology. Additionally, autofluorescence extraction removes background that would otherwise obfuscate minor but important signals. With these capabilities comes the need for proper controls and validation to ensure that the signals that are being recorded are sample-related and not artifacts of preparation, the sample, or the equipment. Increasing governmental regulation of LDT IVD assays under CLIA will be a driving force behind ensuring that the data generated is as controlled and as consistent as possible. Alongside those regulations is a push to increase collaboration across Pharma, CROs, and the academia to share best practices and develop proper programs to support the growth and development of this impactful platform.

Artificial intelligence will play a role in not only allowing for more controlled and carefully collected data but also enabling rapid turnaround so that the massive volumes of information does not slow the development and testing of therapeutics for the patients who will be helped by these advances [137,138,139].

Other potentially transformative technologies in the form of ghost flow, LASE, and imaging cytometry, among others, will provide an expanded set of tools, allowing us to gain a greater understanding of the medicines that are being tested in the clinical space and the diseases they target, to usher in an age of enhanced clinical safety and efficacy. As with SFC, stringent validation will be needed to gain alignment both within trials and from trial to trial. As standardization is achieved, datasets will become more robust and decision-making information more readily available to accelerate the advancement of care.

## Figures and Tables

**Table 1:** Spectral flow cytometry applications in MRD detection across hematologic malignancies.

Disease	SFC Panel Design	MRD Sensitivity	Key Advantages	References
B-ALL	23-color panel	~10^−5^	Enhanced detection of dim antigens; improved separation of leukemic and normal phenotypes	[11,23]
AML	27-color (Roswell Park) and 24-color (Chen et al.) single-tube assays	Down to 0.0132%	High-resolution profiling of blast maturation; single-tube format	[23,28]
CLL and Lymphoma	Panels with CD5, CD19, CD20, CD23, CD79b, ROR1	As low as 0.005%	Increased specificity; improved detection of rare malignant clones	[11,28,29,30]
Multiple Myeloma	EuroFlow NGF markers (CD38, CD138, CD45, CD19, CD56, CD27, CD81, CD117, cytoplasmic κ/λ) in a single-tube assay	~10^−6^	Clear distinction of clonal vs. normal plasma cells; supports standardized MRD endpoints	[31]

**Table 2:** Comparison of fluorochrome families for spectral flow cytometry applications.

Fluorochrome Family	Key Strengths	Reported Limitations
BD RealYellow™ and RealBlue™	✓ Minimal cross-laser excitation ✓ Low background ✓ Suitable for panel expansion	✓ Limited color options
BioLegend Spark Dyes	✓ Narrow emission spectra ✓ Quenching-resistant ✓ Fixation-stable	✓ Moderate brightness ✓ Minor cross-excitation
Bio-Rad StarBright™ Dyes	✓ High brightness ✓ Fixable ✓ Wide spectral range	✓ Not validated for intracellular targets
Biotium CF^®^ Dyes	✓ PEGylation reduces non-specific binding ✓ High solubility	✓ Moderate brightness ✓ Broad excitation range
NovaFluor™ (Thermo Fisher)	✓ Minimal spillover ✓ Unique spectral signatures ✓ Spectral compatibility	✓ Lower brightness ✓ Background staining
BioLegend Fire™ Tandem Dyes	✓ Far-red spectral coverage ✓ Improved tandem stability ✓ Supports high-parameter panels	✓ FRET bleed-through ✓ Cross-laser excitation

**Table 3:** OMIPs utilizing spectral flow cytometry for the analysis of human cellular subsets (updated January 2025).

Number	Title	Reference	Species	Target Cell Type
OMIP-069	OMIP 69, 40-Color Full Spectrum Flow Cytometry Panel for Deep Immunophenotyping of Major Cell Subsets in Human Peripheral Blood	[72]	Human	PBMC
OMIP-084	28-Color Full Spectrum Flow Cytometry Panel for the Comprehensive Analysis of Human γδ T Cells	[31]	Human	γδ T cells
OMIP-097	High-parameter phenotyping of human platelets by spectral flow cytometry	[89]	Human	Platelets
OMIP-099	31-Color Spectral Flow Cytometry Panel to Investigate the Steady-State Phenotype of Human T Cells	[90]	Human	T cells
OMIP-102	50-color phenotyping of the human immune system with in-depth assessment of T cells and dendritic cells	[5]	Human	T cells and dendritic cells
OMIP-109	45-color full spectrum flow cytometry panel for deep immunophenotyping of the major lineages present in human peripheral blood mononuclear cells with emphasis on the T cell memory compartment	[30]	Human	PBMC/T cells

**Table 4:** Groups contributing to standardization in cytometry.

Group/Consortium	Focus	Contributions
EuroFlow Consortium (https://www.euroflow.org)	Hematological diagnostics and MRD detection.	Developed SOPs and uniform antibody panels, automated analysis tools, and ensured interlaboratory reproducibility.
Human Immunology Project Consortium (HIPC) (HIPC DASHBOARD)	Immunophenotyping standardization.	Created 8-color panels, lyophilized reagents, and automated gating tools.
The ONE Study (The ONE Study)	Immune profiling post-organ transplantation.	Designed 7–9-color panels, dried reagents, and guidelines for multicenter variability studies.
European Research Initiative on CLL (ERIC) (ERIC—European Research Initiative on CLL)	MRD detection in CLL.	Developed 4-color and 6-color panels, benchmarked against next-generation sequencing.
Children’s Oncology Group (COG) (childrensoncologygroup.org)	MRD detection in pediatric ALL.	Standardized antibody panels, implemented QA schemes, and provided educational programs.
AIEOP-BFM Group (Leucemia Linfoblastica Acuta—AIEOP)	Pediatric ALL MRD evaluation.	Developed 4-color and 8-color panels aligned with PCR-based methods.
PRECISESADS Project (PRECISESADS IHI Innovative Health Initiative)	Immune monitoring in autoimmune diseases.	Created nine 8-color panels, tested across multiple instruments in multicenter studies.
Canadian National Transplant Research Program (CNTRP) (Transplantation Research Initiative—CIHR)	Immune monitoring for transplant research.	Extended The ONE Study’s findings with automated data analysis pipelines.
Harmonemia Project (Harmonemia: a universal strategy for flow cytometry immunophenotyping-A European LeukemiaNet WP10 study Search Guidelines and other CDMTs Best practices EuroBloodNet EuroBloodNet)	Harmonization of lymphocytosis screening.	Demonstrated compatibility between alternative reagents and different cytometers.
PRECISAIDS (https://doi.org/10.1016/j.autrev.2016.07.034, accessed on 14 June 2025)	Quality assurance and reproducibility.	Published data on interlaboratory variability and standardization in multicenter studies.
CLIP Childhood Leukemia Investigation Program (CLIP—Childhood Leukaemia Investigation Prague 2. lékařská fakulta Univerzity Karlovy)	Pediatric leukemia diagnostics.	Developed guidelines for gating strategies and immunophenotyping.
Swiss Cytometry Society (SCS) (Swiss Cytometry Society Welcome to the World of Flow)	Interlaboratory standardization.	Conducted feasibility studies on EuroFlow panel performance and local QA programs.
SOULCAP (soulcap.org)	Global standardization of immune cell population identification and semantic annotation.	Establishes a consensus on marker combinations and gating strategies, which ensures that immune cell populations are consistently identified across studies and laboratories. Introduces standardized terminologies and data formats. Improves data sharing and reuse. Enhances automation and computational analysis, facilitating translational research.
NIST consortium (NIST Flow Cytometry Standards Consortium NIST)	Development of reference standards and interlaboratory studies to standardize flow cytometry measurements.	Enhances comparability and robustness in cell characterization, supporting applications like CAR-T manufacturing and regulatory approvals by the FDA and EMA.

**Table 5:** Summary of controls in spectral flow cytometry.

Control	Monitored Biological Feature	Application	Source
**Synthetic Controls**			
FlowCytes^®^	Optical and fluorescence properties of lymphocytes, monocytes, granulocytes	Flow cytometry calibration	Slingshot Biosciences, Emeryville, CA, USA
TruCytes™	Biochemical and fluorescence markers of disease states (e.g., AML, HIV)	Disease-specific biomarker detection, assay standardization	
ScatterGrid™	Forward and side scatter visualization through 9 populations seen as a 3x3 grid on forward and side scatter parameter properties (size and granularity) of biological cells	Instrument standardization, calibration	
p24+ T Cell Mimics	p24 antigen expression, mimicking HIV-infected T cells	HIV research, flow cytometry assay control	
Kasumi-3 AML Cell Mimics	Expression of AML markers (CD34, CD117, CD45, CD33)	Acute myeloid leukemia diagnosis and assay validation	
Ki-67 Lymphocyte Mimics	Ki-67 expression as an indicator of cellular proliferation	Rare marker detection, flow cytometry validation	
**Lyophilized PBMC Controls**			
Streck CD-Chex^®^ Plus	T-lymphocytes, B-lymphocytes, NK cells, and CD34+ populations	Immunophenotyping, leukemia/lymphoma quality controls	Streck, La Vista, NE, USA
Streck CD-Chex^®^ TdT Plus	TdT, CD1a, CD34, cytoplasmic CD3	Rare antigen detection in hematopoietic neoplasms	
Streck CD-Chex^®^ CD117 Plus	CD117, CD25, CD71 markers	Immunophenotyping, detection of abnormal leukocyte populations	
ClearLLab™ Control Cells	Leukocyte populations for myeloid and lymphoid lineages	Immunophenotyping, leukemia/lymphoma diagnostics	Beckman Coulter, Brea, CA, USA
BD Biosciences Controls	Lymphocyte and myeloid populations	Routine QC, instrument calibration	BD Biosciences, San Jose, CA, USA
Veri-Cells™	T cells, B cells, and natural killer cells	Routine QC, reproducibility in multicenter and longitudinal studies	BioLegend, San Diego, CA, USA

**Table 6:** Unmixing models.

Unmixing Algorithm	Overview	Advantages	Limitations
Ordinary Least Squares (OLS) [109]	Assumes linear combinations, constant noise across detectors.	Fast computation; good for high-intensity signals.	Sensitive to variable noise; may produce negative values; poor for dim markers.
Non-Negative Least Squares (NNLS) [1]	Constrains OLS solutions to non-negative values.	Prevents negative intensities; robust for moderate signals.	May zero out weak true signals, reducing sensitivity.
Weighted Least Squares (WLS) [1]	Applies weights to detectors based on noise level.	Improved dim marker resolution; accounts for heteroscedasticity.	Requires accurate noise estimation; more complex.
Poisson Regression-Based Unmixing [1]	Models photon counting noise (variance proportional to mean).	Superior preservation of low-intensity signals; best biological match.	Computationally intensive; slower on large datasets.
Variance-Stabilizing Transformation (VST) [1]	Stabilizes variance before linear unmixing.	Simplifies unmixing; efficient for moderate noise correction.	Approximate; depends on transformation quality.

**Table 7:** Comparative summary of AF handling features.

Platform/Tool	AF Detection	Multi-AF Support	Integration	Key Strengths	Key Limitations
Sony ID7000 (AF Finder)	Semi-automated wizard with gating	Full (multiple profiles per sample)	Native (on-instrument)	Highly automated; fine spectral gating; excellent resolution in high-AF tissues	Exclusive to Sony; requires good unstained controls; learning curve for new users
Cytek Aurora (AF Explorer)	Semi-automated (via SpectroFlo wizard)	Full (multiple profiles per sample)	Native (SpectroFlo^®^ version 2.2 (or later))	Proven tool; community support; solid AF subtraction, excellent resolution in high-AF tissues; used in multiple publications	Manual subset gating for AF; lacks multi-dimensional gating UI
BD FACSymphony A5 SE	Semi-automated (unmixing via BD FACSDiva™ software version 9.3 (or later))	Partial (global AF only)	Native (FACSDiva™ software version 9.3 (or later)) + FlowJo™ v10.8 (or later)	Compatible with FlowJo re-unmixing; can subtract single AF component	Limited support for multi-AF; less documentation; fewer published workflows
Thermo Fisher Bigfoot	Semi-automated (real-time in software)	Unknown (likely global AF only)	Native (Sasquatch™ Software version 1.x)	First spectral sorter; real-time AF subtraction; broad detection range	Limited documentation; unclear multi-AF handling; software not fully optimized
Miltenyi (MACSQuant)	Manual only (no spectral unmixing)	None	Conventional (non-spectral)	Simple UI; good for low-AF applications	No AF subtraction; gating workaround needed for high-AF samples
Beckman Coulter (CytoFLEX)	Manual only	None	Conventional (non-spectral)	Sensitive detectors; basic AF correction possible via background subtraction	No spectral unmixing; not suitable for high-AF or dim-marker separation
Open-Source/3rd Party	Manual/custom scripts (R, FlowJo, etc.)	Possible (user-defined only)	Post-acquisition only	Flexible; supports novel workflows; FlowJo/FCS Express unmixing available	Requires expertise; no automation; no GUI “AF finder”; not real-time

**Table 8:** Advanced computational tools offer potential for improving spectral unmixing and performing quality assessment and data visualization.

Category	Tools	Purpose	Considerations
Quality Assessment and Data Cleaning	FlowAI, FlowCut, FlowClean, PeacoQC [61,116,117,118,119]	Detect and handle abrupt events during data acquisition, aiding in quality assurance.	Useful for minimizing noise and artifacts in raw data.
Debris and Doublet Removal	FlowSOM, X-Shift, Phenograph [92,118,120]	Identify and exclude debris and doublets to ensure accurate analysis of cell populations.	Effective for refining datasets but requires careful parameter optimization.
Dimensionality Reduction	UMAP t-SNE [121,122]	Enable visualization of high-dimensional data in 2D or 3D for easier interpretation.	Risk of oversimplification, potential for misclassification, and loss of biological detail [123].
Batch Effect Correction	CytoNorm, CytofBatchAdjust, SwiftReg [62,124,125]	Address inter-sample variability to enable consistent analysis across batches.	CytoNorm may introduce artifacts, such as altered CD4/CD8 profiles [21].
Cluster Annotation	Marker Enrichment Modeling [126]	Assign phenotype scores to clusters based on their relative expression profiles to each other.	Absolute expression patterns can be missed (i.e., a MEM annotation will never be CD45+ on leukocytes as all clusters will have this). The annotation is dependent on clustering quality [127].
Sample Comparisons	Tracking Responders Expanding [128]	Pairwise comparisons of dimensionality reduction maps with clustering outputs to determine “hotspots” of phenotypic differences between samples.	Pairwise comparisons will require significant computing power when large sample groups are used. Each sample needs to be subsampled to the same number of cells for meaningful hotspot identification.

## Data Availability

Not applicable.

## Abbreviations

The following abbreviations are used in this manuscript:

AF	Autofluorescence
AIEOP-BFM	Associazione Italiana di Ematologia e Oncologia Pediatrica—Berlin-Frankfurt-Münster
AI	Artificial Intelligence
AML	Acute Myeloid Leukemia
B-ALL	B-cell Acute Lymphoblastic Leukemia
CAP	College of American Pathologists
CAS	CytekAssaySetting
CFC	Conventional Flow Cytometry
CLL	Chronic Lymphocytic Leukemia
CLSI	Clinical and Laboratory Standards Institute
CNTRP	Canadian National Transplant Research Program
COG	Children’s Oncology Group
CRO	Contract Research Organization
CSI	Cross-Stain Index
CS&T	Cytometer Setup and Tracking
DOAJ	Directory of Open-Access Journals
EMA	European Medicines Agency
ERIC	European Research Initiative on CLL
FDA	U.S. Food and Drug Administration
FISH	Fluorescence In Situ Hybridization
FMM/FM-X	Fluorescence-Minus-Multiple
FMO	Fluorescence-Minus-One
HIPC	Human Immunology Project Consortium
ISO	International Organization for Standardization
IVDR	In Vitro Diagnostic Medical Device Regulation
LDT	Laboratory-Developed Test
LDGs	Low-Density Granulocytes
M-MDSCs	Monocytic Myeloid-Derived Suppressor Cells
MDPI	Multidisciplinary Digital Publishing Institute
ML	Machine Learning
MRD	Measurable Residual Disease
NIST	National Institute of Standards and Technology
NMF-RI	Non-Negative Matrix Factorization rank initialization
NNLS	Non-Negative Least Squares
OLS	Ordinary Least Squares
OMIP	Optimized Multicolor Immunophenotyping Panel
PBMCs	Peripheral Blood Mononuclear Cells
pDCs	Plasmacytoid Dendritic Cells
QC	Quality Control
SCRMs	Single-Color Reference Materials
SFC	Spectral Flow Cytometry
SIR	Stain Index Reduction
SOULCAP	Standardization of Immune Cell Population Identification and Semantic Annotation
SRCs	Spectral Reference Controls
SSI	Single Stain Index
SSM	Spillover Spread Matrix
t-SNE	t-Distributed Stochastic Neighbor Embedding
TREX	Tracking Responders Expanding
Tregs	Regulatory T Cells
UMAP	Uniform Manifold Approximation and Projection
VST	Variance-Stabilizing Transformation
WLS	Weighted Least Squares
